# Intravenous Thrombolysis Combined With Endovascular Treatment Versus Endovascular Treatment Alone in Acute Ischemic Stroke due to Tandem Lesions: A Systematic Review and Meta‐Analysis

**DOI:** 10.1002/brb3.71628

**Published:** 2026-07-28

**Authors:** Wenqian Wei, Chongxu Zhong, Jian Tang, Liufei Chen, Kunlong Yao, Liyong Li, Shengliang Shi, Yueling Zhang, Jian Zhang

**Affiliations:** ^1^ Department of Neurology The Second Affiliated Hospital of Guangxi Medical University Nanning Guangxi China

**Keywords:** acute ischemic stroke, bridging therapy, endovascular treatment, intravenous thrombolysis, mechanical thrombectomy, tandem lesions, tandem occlusion

## Abstract

**Objective:**

For patients suffering from acute ischemic stroke (AIS) brought on by tandem lesions (TL), to compare the safety and efficacy of intravenous thrombolysis (IVT) in conjunction with endovascular therapy (EVT) to EVT alone.

**Methods:**

Relevant studies published up until October 20, 2024, were found by a systematic assessment of the literature from the PubMed, Embase, and Cochrane databases. Results such as the 90‐day modified Rankin Scale (mRS) score, effective recanalization, symptomatic intracerebral hemorrhage (sICH), and 90‐day mortality were the main focus of the comparison between IVT plus EVT and EVT alone in patients with TL.

**Results:**

A total of 1756 patients from nine studies were included in the final analysis; 976 of these patients were categorized as IVT + EVT group patients, and 780 as EVT alone group patients. The IVT plus EVT group was associated with a higher rate of successful recanalization (modified Thrombolysis in Cerebral Infarction score [mTICI] ≥ 2*b*/3) (RR = 1.11; 95% CI, 1.04–1.18; *p* < 0.05; *I* ^2^ *= *27%) and improved functional independence at 90 days (mRS 0–2) (RR = 1.27; 95% CI, 1.13–1.42; *p* < 0.05; *I* ^2^ *= *0%). There was no discernible difference in the two groups' risk of sICH (RR = 0.72; 95% CI, 0.46–1.12; *p* > 0.05; *I* ^2^ *= *20%). Furthermore, the 90‐day mortality rate was lower for IVT with EVT than for EVT alone (RR = 0.67; 95% CI, 0.52–0.87; *p* < 0.05; *I* ^2^ *= *0%).

**Conclusion:**

IVT + EVT, as opposed to EVT alone, may be correlated with superior functional results at 90 days in AIS patients with TL.

## Graphical abstract

Our systematic review and meta‐analysis evaluate whether intravenous thrombolysis (IVT) plus endovascular therapy (EVT) outperforms EVT alone in patients with acute ischemic stroke due to tandem lesions. Pooled data from nine studies involving 1756 patients demonstrate that IVT + EVT is associated with significantly higher rates of functional independence (modified Rankin Scale score 0–2) at 90 days and successful recanalization. Importantly, the IVT + EVT approach also yielded a lower mortality risk without increasing the rate of symptomatic intracranial hemorrhage compared to EVT alone.

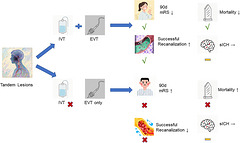

## 1 Introduction

The majority of strokes, about 87%, are ischemic strokes, which contribute significantly to both disability and death (Saini et al. 2021). About 10%–20% of instances are tandem lesions (TL), which are a challenging subtype of large‐vessel occlusion (LVO) stroke, typically characterized by severe extracranial internal carotid artery (ICA) stenosis or occlusion with ipsilateral intracranial occlusion. Compared with isolated intracranial occlusion, TL are associated with worse functional outcomes and more complex reperfusion strategies (Wilson et al. 2018).

Endovascular treatment (EVT) has become the standard reperfusion therapy for eligible patients with anterior‐circulation LVO, while intravenous thrombolysis (IVT) remains recommended for patients who meet treatment criteria and have no contraindications. Current guidelines further emphasize that IVT should not delay EVT in patients eligible for both therapies (Turc et al. 2023; Berge et al. 2021; Powers et al. 2019). Studies have shown the safety and efficacy of EVT in TL (Anadani et al. 2021; Assis et al. 2018). However, the optimal role of bridging therapy, defined as IVT followed by EVT, remains uncertain in the specific setting of TL. Theoretical advantages of bridging therapy include early partial reperfusion, thrombus softening, and improved distal embolus clearance, whereas concerns persist regarding hemorrhagic risk, the need for periprocedural antiplatelet therapy, and the technical complexity of acute carotid intervention.

At present, the evidence base for TL is dominated by retrospective observational studies and registry‐based analyses, and randomized controlled trials (RCTs) data specifically addressing this population are lacking. Previous systematic reviews and meta‐analyses have suggested that bridging therapy may be associated with improved functional outcomes without a clearly increased risk of symptomatic intracranial hemorrhage (sICH) (Anadani et al. 2022; Marnat et al. 2021; Mujanovic et al. 2024; Rodriguez‐Calienes et al. 2023). However, the certainty of these findings is limited by heterogeneous definitions of TL, variability in IVT agents and endovascular strategies, inconsistent outcome definitions, and the potential for selection bias and confounding by indication.

Therefore, we conducted an updated systematic review and meta‐analysis to compare IVT plus EVT with EVT alone in patients with acute ischemic stroke (AIS) due to TL. We aimed to summarize the available observational evidence regarding functional outcomes, recanalization, mortality, and sICH, and to identify remaining evidence gaps relevant to clinical decision‐making.

## 2 Methods

### 2.1 Protocol and Guidance

The Preferred Reporting Items for Systematic Reviews and Meta‐Analyses (PRISMA) guidelines were consulted in the preparation of this meta‐analysis.

### 2.2 Eligibility Criteria

Relevant papers were found using the PICOS (Patient population, Intervention, Control, Outcome, Study design) framework, which is a popular approach in evidence‐based medicine: (1) the patient population consisted of individuals aged ≥ 18 years diagnosed with TL. In this review, TL are defined as acute anterior‐circulation LVO associated with ipsilateral severe stenosis or occlusion of the extracranial carotid artery, most commonly the cervical ICA. Intracranial occlusions generally include the intracranial ICA, M1 segment, or M2 segment of the middle cerebral artery (MCA). Given the heterogeneity in stenosis thresholds across studies, extracranial carotid stenosis of > 70% or occlusion was considered the working definition of TL, while the original definition used in each included study was extracted and reported separately (included in Table S2); (2) the intervention under study was IVT plus EVT, which included patients who received IVT before EVT; (3) the control group was EVT alone, which included patients who did not receive IVT before EVT; (4) the study assessed a number of outcomes, including functional outcomes, successful recanalization, mortality at 90 days, and sICH; (5) various definitions of sICH were accepted in the investigation. The extracranial carotid lesion was typically identified on baseline computed tomography angiography, magnetic resonance angiography, digital subtraction angiography, or angiographic assessment during the endovascular procedure. In emergency practice, exact stenosis quantification may be difficult before reperfusion therapy; therefore, some studies relied on procedural angiographic confirmation. We extracted and accepted the definitions reported by each original study. Patients were chosen for EVT treatment according to the participating center's procedure; there was no set selection process for EVT treatment.

### 2.3 Information Sources and Search Strategy

We systematically searched PubMed, Embase, and the Cochrane Library from database inception to October 20, 2024, without regard to language restrictions. The search strategy combined controlled vocabulary and free‐text terms related to AIS, TL or tandem occlusions, EVT or mechanical thrombectomy, and IVT or bridging therapy. Reference lists of included studies and relevant reviews were manually screened. The detailed search strategies for each database are provided in the Supporting Information.

### 2.4 Study Selection

To ascertain eligibility, three authors (C.X.Z., J.Z., and W.Q.W.) independently examined the abstracts and titles of the reports that were retrieved. The entire texts of articles that might be relevant were then evaluated. Any disagreements about judgment were settled by consensus. We did not include abstracts, unpublished studies, gray literature, reviews, meta‐analyses, case reports or series with less than six individuals, and case series without an IVT plus EVT group because of the limited availability of peer‐reviewed data, incomplete outcome reporting, and difficulty in assessing methodological quality. RCTs and observational cohort studies comparing IVT plus EVT with EVT alone were considered eligible. However, no RCTs specifically addressing patients with AIS due to TL were identified. All included studies were retrospective observational studies. Excluded from the analysis were studies with no data on our outcomes of interest and duplicate patient data supplied by other research.

### 2.5 Data Extraction

For every qualifying study, three authors (C.X.Z., J.Z., and W.Q.W.) separately retrieved data on baseline information, functional outcomes, and safety outcome indicators: (1) general information, including the names of the authors, the research type, the year of publication, and the demographics of the patients; (2) functionally independent patients are those who score 0–2 on the 90‐day modified Rankin Scale (mRS); (3) successful recanalization is defined as a modified Thrombolysis in Cerebral Infarction (mTICI) score of 2*b*/3, as reported by the original studies, generally assessed on the final angiogram after the endovascular procedure unless otherwise specified; (4) 90‐day mortality and sICH are the safety outcomes. sICH was defined according to the criteria reported in each original study, including ECASS II, ECASS III, SITS‐MOST, or other study‐specific definitions. Additional safety outcomes, including any intracranial hemorrhage, extracranial bleeding, parenchymal hematoma, and procedure‐related complications, were extracted when available. We extracted the definition of TL used in each included study, including whether extracranial ICA occlusion, severe stenosis, or both were included, and whether the intracranial occlusion involved the distal ICA, MCA, or both (included in Table S2).

### 2.6 Risk of Bias Assessment

Two authors (W.Q.W. and C.X.Z.) reviewed the literature, and the Newcastle‐Ottawa Scale (NOS) for observational studies was used to gauge the studies's methodological quality.

### 2.7 Heterogeneity Analysis

We planned to explore heterogeneity using subgroup analyses according to age, baseline National Institutes of Health Stroke Scale (NIHSS) score, Alberta Stroke Program Early CT Score (ASPECTS), occlusion site, intravenous thrombolytic agent, and endovascular strategy if sufficient study‐level data were available. However, these analyses could not be reliably performed because the relevant variables were inconsistently reported and the number of included studies was limited.

### 2.8 Synthesis Methods

RevMan 5.4 from the Cochrane Collaboration Network was used for the meta‐analysis. A cumulative meta‐analysis of the included studies’ functional and safety results was carried out. Because relative estimates for the relative risk (RR) were similar across trials with different designs, populations, and follow‐up lengths, we chose RR as the effect measure. The RR was estimated at a 95% confidence interval using binary data from published studies and then meta‐analyzed using the Mantel–Haenszel random effects model. The Mantel–Haenszel approach took into account sample size and event rates when it was used. At a significance threshold of *p* ≤ 0.05, the observed difference was determined to be statistically significant. The *χ* ^2^ test and Higgins index (*I* ^2^) were used to assess data heterogeneity. An *I* ^2^ result of more than 50% indicates significant heterogeneity.

## 3 Results

### 3.1 Literature Review and Study Selection

The PRISMA criteria were followed for conducting the systematic review and meta‐analysis. A total of 761 items were initially determined to be possibly pertinent. After removing duplicates and screening titles and abstracts to eliminate irrelevant research, case reports, and reviews, nine papers (Anadani et al. 2022; Marnat et al. 2021; Mujanovic et al. 2024; Rodriguez‐Calienes et al. 2023; Grigoryan et al. 2016; Heck and Brown 2015; Pikija et al. 2019; Sallustio et al. 2017; Sanak et al. 2023) met all inclusion criteria. The details of our literature search are available in the Supporting Information of Table S1 and Figure S1.

### 3.2 Study Characteristics

The final study comprised data from nine investigations, totaling 1756 patients, of whom 976 (56.0%) received IVE plus EVT treatment. Table 1 lists critical factors for each study included in the meta‐analysis: study author, year of publication, country, study design, sex ratio, the ASPECTS (ASPECTS is used to evaluate the early ischemic changes in the MCA blood supply area of AIS patients, and it is an imaging evaluation index), and the median NIHSS (NIHSS is used to assess the degree of neurological impairment in stroke patients). No study found any significant differences between the two groups for other known predictors, such as baseline NIHSS, ASPECTS, atrial fibrillation, diabetes, or hypertension. Table 1 displays the attributes of the patients who were included.

**TABLE 1:** Details of included studies.

Study	Country	Study design	Median age	Male(%)	ASPECTS	Median NIHSS
Anadani, 2022 (Anadani et al. 2022)	Worldwide	R	62.1 ± 12.8 vs. 64.3 ± 13.3	69.5 vs. 64.9	8 vs. 7	16 vs. 16
Grigoryan, 2016 (Grigoryan et al. 2016)	USA	R	64.4 ± 12.5	64	7.5 ± 1.6	17.6 ± 5.0
Heck, 2015 (Heck and Brown 2015)	USA	R	69.5	NA	7.7	17
Marnat, 2021 (Marnat et al. 2021)	Worldwide	R	51.9 ± 11.0 vs. 52.0 ± 12.9	66.7 vs. 66	7 vs. 7	15 vs. 17
Mujanovic, 2024 (Mujanovic et al. 2024)	Worldwide	R	69	73	8	19
Pikija, 2019 (Pikija et al. 2019)	Worldwide	R	75.9	52.8	NA	14 vs. 16
Rodriguez‐Calienes, 2023 (Rodriguez‐Calienes et al. 2023)	Worldwide	R	66 vs. 68	71.1 vs. 69	9 vs. 8	17 vs. 15
Sallustio, 2017 (Sallustio et al. 2017)	Italy	R	65.6 ± 12.8	61.1	6.9 ± 2.28	19 ± 2.9
Sanak, 2023 (Sanak et al. 2023)	Worldwide	R	68.4 ± 10.9 vs. 69.2 ± 12.8	64.1 vs. 50.8	15 vs. 17	NA

Abbreviation: R = retrospective.

### 3.3 Risk of Bias in the Included Studies

As shown in Table 2, the NOS scale was used to evaluate the observational studies that were part of this research. The evaluation of publication bias and the financing sources of the included publications, however, were not sufficiently discussed. Currently, the NOS scale lacks a regulated quality grading mechanism. Studies with seven or more stars on the NOS scale were considered to be of high quality, in line with other research (Yuhara et al. 2011). The nine observational studies that were part of this analysis had an average NOS score of 7.8 stars. Consequently, it was decided that the observational studies that were part of this analysis were of high quality.

**TABLE 2:** Authors judgment of the quality of observational studies.

	Selection				Comparability	Outcome			
Study	Representativeness of the exposed cohort	Selection of the non exposed cohort	Ascertainment of exposure	Demonstration that outcome of interest was not present at start of study	Comparability of cohorts on the basis of the design or analysis	Assessment of outcome	Was follow‐up long enough for outcomes to occur	Adequacy of follow up of cohorts	Total scores
Anadani, 2022 (Anadani et al. 2022)	**✵**	**✵**	**✵**	**✵**	**✵✵**	**✵**	**✵**	**✵**	9
Grigoryan, 2016 (Grigoryan et al. 2016)	**✵**	**✵**	**✵**	**✵**		**✵**	**✵**	**✵**	7
Heck, 2015 (Heck and Brown 2015)	**✵**	**✵**	**✵**	**✵**		**✵**	**✵**	**✵**	7
Marnat, 2021 (Marnat et al. 2021)	**✵**	**✵**	**✵**	**✵**	**✵✵**	**✵**	**✵**	**✵**	9
Mujanovic, 2024 (Mujanovic et al. 2024)	**✵**	**✵**	**✵**	**✵**		**✵**	**✵**	**✵**	7
Pikija, 2019 (Pikija et al. 2019)	**✵**	**✵**	**✵**	**✵**		**✵**	**✵**	**✵**	7
Rodriguez‐Calienes, 2023 (Rodriguez‐Calienes et al. 2023)	**✵**	**✵**	**✵**	**✵**	**✵✵**	**✵**	**✵**	**✵**	9
Sallustio, 2017 (Sallustio et al. 2017)	**✵**	**✵**	**✵**	**✵**		**✵**	**✵**	**✵**	7
Sanak, 2023 (Sanak et al. 2023)	**✵**	**✵**	**✵**	**✵**	**✵✵**	**✵**	**✵**	**✵**	9
Mean									7.8

*Note*: “**✵**” denotes a score of 1 point.

### 3.4 EVT Effect Meta‐Analysis

#### 3.4.1 Functional Outcomes

There were 976 patients in the IVT plus EVT group and 780 patients in the EVT only group. IVT plus EVT was significantly associated with an increase in functional independence (mRS 0–2) (RR = 1.27; 95% CI, 1.13–1.42; *p* < 0.05; *I* ^2^ = 0%) in the primary outcome analysis, which showed that 474 out of 976 patients (48.6%) in the IVT plus EVT group attained functional independence (Figure S2A). The IVT + EVT group fared better than the EVT alone group in the secondary outcome analysis (mTICI ≥ 2*b*/3)(RR = 1.11; 95% CI, 1.04–1.18; *p* < 0.05; *I* ^2^ = 27%) (Figure S2B). The results showed that higher recanalization rates and improved functional outcomes might be linked to IVT before EVT.

#### 3.4.2 Safety Outcomes

Within the 90‐day period, 208 deaths (11.8%) occurred among the 1756 patients that were part of the trial. The IVT plus EVT group had a lower 90‐day mortality rate than the EVT alone group, according to the results. In other words, IVT prior to EVT did not raise the 90‐day mortality rate for patients with TL (RR = 0.67; 95 % CI, 0.52–0.87; *p* < 0.05; *I* ^2^ = 0%). There was no statistically significant difference in sICH between the IVT plus EVT group and the EVT alone group. (RR = 0.72; 95% CI, 0.46–1.12; *p* > 0.05; *I* ^2^ = 20%) (refer to Figure S2C,D for further details). Additional bleeding outcomes, including any intracranial hemorrhage, extracranial bleeding, and parenchymal hematoma, were not consistently reported across the included studies and therefore could not be quantitatively synthesized.

## 4 Discussion

Our findings suggest that IVT plus EVT in patients with TL may be associated with better functional outcomes, higher reperfusion rates, and lower mortality rates than EVT alone, and does not raise the risk of sICH.

Previous research has demonstrated that EVT alone is not inferior to IVT with EVT (Yang et al. 2020; Suzuki et al. 2021), but these studies either had limited representation or omitted individuals with TL. Currently, there is considerable debate in clinical practice about whether IVT should be used before EVT. This is because patients with TL frequently require combination antiplatelet therapy following carotid artery stenting, and the use of IVT is thought to increase the risk of bleeding and delay arterial puncture, thereby exacerbating poor functional outcomes (Menon et al. 2016). However, in our investigation, IVT combined with EVT treatment for TL patients was associated with superior functional results, higher reperfusion rates, and decreased mortality. Anadani et al. (2022) and Pikija et al. (2019) also imply that IVT is a predictor of better outcomes and higher recanalization rates in TL patients, which is consistent with our research. TL patients frequently have a higher thrombus load, and IVT plays a crucial role in dissolving the clot and attaining distal reperfusion. We believe that IVT can swiftly commence the thrombolytic process, reduce thrombus load, and improve circumstances for later EVT. In addition, IVT can dissolve difficult‐to‐reach clots and extra microemboli (Pikija et al. 2019). The infarct size may increase as a result of the complex vascular lesions and extended recanalization time, particularly in individuals with TL. Nonetheless, IVT is linked to a favorable functional outcome and improves early reperfusion even when intravascular treatment is postponed by more than 30 min (Zhou et al. 2022). In addition, Lin's et al. (2023) study demonstrated that bridging IVT before EVT was beneficial in clearing the thrombotic load and reducing the reperfusion duration in stroke patients whose infarct core expanded rapidly within 4.5 h. The fact that the size of the thrombus recovered following bridging therapy is substantially smaller than that following EVT alone may potentially contribute to the increased success rate of IVT plus EVT recanalization (Rossi et al. 2022). Reduced mortality and functional recovery are directly correlated with higher reperfusion rates because they can lessen the infarction area's growth.

In addition, arterial recanalization and positive functional outcomes following thrombolysis are independently linked to favorable collateral status (Liebeskind et al. 2014). By administering thrombolytic medications to both ends of the well‐branching thrombus, IVT is thought to promote early vascular recanalization, which is favorable for a good prognosis (Seners et al. 2019). Nevertheless, there is little data to support the idea that an IVT alone can encourage the development of collateral circulation prior to EVT. Adjuvant antiplatelet treatment and carotid stenting may significantly aid in the development of collateral circulation in TL. Thus, researchers (Liu et al. 2023) have conjectured whether bridging therapy successfully enhances blood flow to injured brain tissue through collateral circulation, thereby improving clinical prognosis, and whether patients with TL have better collateral circulation than patients with isolated lesions. To validate these theories, more investigation is required.

IVT prior to EVT does not raise the risk of sICH, according to our research. However, there is a theoretical risk of bleeding because patients undergoing carotid stenting with TL must also receive antiplatelet therapy. According to studies, bleeding risk may not only be correlated with IVT but also with age and the treatment time window. The primary reason for not using IVT may not be bleeding (Hu et al. 2024). Thus, more RCTs are required to investigate how IVT and antiplatelet treatment interact during stenting. Information on thrombolytic agent type, dose, and infusion timing was incompletely reported across studies. Most studies reported intravenous alteplase as the primary thrombolytic agent, whereas data on tenecteplase or other agents were limited or unavailable, limiting interpretation of how the thrombolytic regimen may influence efficacy and safety. Important safety outcomes, such as any intracranial hemorrhage, extracranial bleeding, parenchymal hematoma, and access‐site complications, were not uniformly reported. Consequently, our safety analysis was mainly limited to sICH and 90‐day mortality, which may underestimate the overall bleeding burden associated with bridging therapy.

Current stroke guidelines recommend IVT for eligible patients with AIS presenting within the approved therapeutic time window and EVT for eligible patients with anterior‐circulation LVO. For patients eligible for both treatments, guidelines generally recommend that IVT should not delay EVT. However, specific recommendations for TL stroke remain limited because these patients were underrepresented in pivotal randomized trials and because the optimal management of the extracranial carotid lesion remains uncertain. Our findings are broadly consistent with guideline principles supporting IVT in otherwise eligible patients before EVT, but they should not be interpreted as definitive evidence specific to TL. Individualized decision‐making remains necessary, particularly in patients requiring acute carotid stenting (ACS), antiplatelet therapy, or those with high bleeding risk (Turc et al. 2023; Berge et al. 2021; Powers et al. 2019).

Baseline imbalances between the IVT plus EVT group and the EVT alone group were present in some included studies, including differences in age, baseline stroke severity, ASPECTS, vascular risk factors, onset‐to‐treatment time, and eligibility for thrombolysis. Because the meta‐analysis was based mainly on aggregate unadjusted data, we could not consistently adjust for these confounders across studies. Although some original studies performed multivariable adjustment or propensity‐score analyses, the adjustment variables were heterogeneous. Therefore, residual confounding may have influenced the observed association between treatment strategy and outcomes. The timing and method used to determine extracranial carotid stenosis varied among studies. In the hyperacute setting, precise measurement of 70%–99% stenosis before EVT may be challenging because of near‐occlusion, poor distal flow, thrombus burden, and time constraints. Some lesions may have been confirmed or reclassified during digital subtraction angiography. This variability may have led to misclassification of TL and should be considered when interpreting the results. We could not perform subgroup analyses comparing extracranial ICA occlusion with severe stenosis because most studies did not provide outcome data separately for these two anatomical subgroups. This distinction may be clinically relevant because complete occlusion and severe stenosis may differ in procedural complexity, collateral status, need for ACS, and hemorrhagic risk.

Several limitations should be acknowledged. First, all included studies were retrospective observational studies, and no RCTs specifically focusing on TL stroke were available. Therefore, the pooled estimates should be interpreted as associations rather than causal effects. Because treatment allocation was not randomized, patients who received or did not receive IVT before EVT may have differed in baseline stroke severity, infarct core size, treatment time windows, contraindications to thrombolysis, vascular risk factors, collateral status, and planned endovascular strategy. These differences may have introduced selection bias, confounding by indication, and residual confounding.

Second, EVT strategies were not standardized across the included studies. Treatment decisions were generally made by the treating stroke center or interventionalist after clinical and imaging evaluation. Thus, differences in thrombectomy technique, ACS or angioplasty, antiplatelet or anticoagulation regimens, and procedural sequencing may have influenced reperfusion, hemorrhagic complications, functional outcomes, and mortality. However, the relevant procedural details were not consistently reported, preventing further assessment of whether specific EVT approaches modified the effect of IVT + EVT versus EVT alone.

Third, clinical and methodological heterogeneity existed across studies, including differences in patient selection, definitions of TL, thrombolytic regimens, treatment time windows, imaging protocols, outcome definitions, and follow‐up assessments. Although random‐effects models were used, residual heterogeneity could not be fully excluded. In addition, the number of studies and patients was limited for some outcomes, particularly sICH and reperfusion‐related outcomes, which may have reduced the precision of the pooled estimates.

Finally, this meta‐analysis was based on aggregate published data rather than individual patient‐level data. Therefore, adjusted analyses using uniform covariates and detailed subgroup analyses according to occlusion site, cervical lesion characteristics, infarct core volume, collateral status, stroke severity, treatment timing, thrombolytic agent, antiplatelet use, and EVT strategy could not be performed. Publication bias also could not be completely excluded. Accordingly, although our results suggest a potential benefit of IVT + EVT in patients with TL stroke, well‐designed prospective studies and RCTs are needed to provide more conclusive evidence.

## 5 Conclusions

According to a pooled review of available data, IVT plus EVT in patients with TL may be linked to better 90‐day functional outcomes, higher recanalization rates, and lower mortality than EVT alone. It also does not raise the risk of sICH. However, because all included studies were retrospective and non‐randomized, these findings should be interpreted cautiously and require confirmation in prospective studies and RCTs.

## Author Contributions

**Wenqian Wei**: conceptualization (lead), writing – original draft (lead), formal analysis (lead), writing – review and editing (equal). **Chongxu Zhong**: methodology (lead), software (lead), writing – review and editing (equal). **Jian Tang**: formal analysis (supporting), writing – review and editing (equal). **Liufei Chen**: conceptualization (supporting), writing – review and editing (equal). **Kunlong Yao**: writing – original draft (supporting), writing – review and editing (equal). **Liyong Li**: software (supporting), writing – review and editing (equal). **Shengliang Shi**: writing – original draft (supporting), writing – review and editing (equal). **Yueling Zhang**: writing – review and editing (equal). **Jian Zhang**: writing – review and editing (equal).

## Funding

The authors have nothing to report.

## Ethics Statements

No ethical approval was sought because only data from previously published studies in which informed consent was obtained were retrieved and analyzed.

## Conflicts of Interest

The authors declare no conflicts of interest.

## Supporting information




**Supporting Information**: brb371628‐sup‐0001‐SuppMat.pdf

## Data Availability

The data supporting the findings of this study are derived from previously published studies included in this systematic review and meta‐analysis. All data analyzed in this study are available within the original published articles and their supplementary materials. Additional data extracted or generated during the current study are available from the corresponding author upon reasonable request.
