# Supplementary material for: Intravenous Thrombolysis Combined With Endovascular Treatment Versus Endovascular Treatment Alone in Acute Ischemic Stroke due to Tandem Lesions: A Systematic Review and Meta‐Analysis
Source: Brain Behav. 2026 Jul 28;16(8):e71628. doi: 10.1002/brb3.71628 (PMC13411295; doi:10.1002/brb3.71628)
Supplement: Supplementary file 1 — Supporting Information: brb371628‐sup‐0001‐SuppMat.pdf [file BRB3-16-e71628-s001.pdf]

Intravenous thrombolysis combined with endovascular treatment versus endovascular treatment alone in acute ischemic stroke due to tandem lesions: a systematic review and meta-analysis

Table S1 Search strategies

Database	Step	Search algorithm	Items found
Pubmed	#1	"Stroke"[Mesh] OR "Brain Ischemia"[Mesh] OR "Ischemic Stroke"[Title/Abstract] OR "Ischaemic Stroke"[Title/Abstract] OR "Acute Ischemic Stroke"[Title/Abstract] OR "Acute Ischaemic Stroke"[Title/Abstract] OR "Cerebral Infarction"[Title/Abstract] OR "Large Vessel Occlusion"[Title/Abstract] OR "LVO"[Title/Abstract]	296184
	#2	"tandem lesion"[Title/Abstract] OR "tandem lesions"[Title/Abstract] OR "tandem occlusion"[Title/Abstract] OR "tandem occlusions"[Title/Abstract] OR "tandem stenosis"[Title/Abstract] OR "tandem stenoses"[Title/Abstract] OR "tandem pathology"[Title/Abstract] OR "cervical carotid occlusion"[Title/Abstract] OR "cervical carotid stenosis"[Title/Abstract] OR "extracranial carotid occlusion"[Title/Abstract] OR "extracranial carotid stenosis"[Title/Abstract] OR "cervical internal carotid artery"[Title/Abstract] OR "extracranial internal carotid artery"[Title/Abstract] OR "Carotid Arteries"[Mesh] OR "Carotid Stenosis"[Mesh] OR "Carotid Artery Thrombosis"[Mesh]	79810
	#3	"Thrombectomy"[Mesh] OR "Endovascular Procedures"[Mesh] OR "thrombectomy"[Title/Abstract] OR "mechanical thrombectomy"[Title/Abstract] OR "endovascular treatment"[Title/Abstract] OR "endovascular therapy"[Title/Abstract] OR "endovascular reperfusion"[Title/Abstract] OR "endovascular intervention"[Title/Abstract] OR "EVT"[Title/Abstract] OR "MT"[Title/Abstract] OR "stent retriever"[Title/Abstract] OR "aspiration thrombectomy"[Title/Abstract] OR "carotid artery stenting"[Title/Abstract] OR "carotid stenting"[Title/Abstract] OR "stenting"[Title/Abstract]	293873
	#4	"Thrombolytic Therapy"[Mesh] OR "Tissue Plasminogen Activator"[Mesh] OR "thrombolysis"[Title/Abstract] OR "intravenous thrombolysis"[Title/Abstract] OR "IVT"[Title/Abstract] OR "bridging therapy"[Title/Abstract] OR "bridging thrombolysis"[Title/Abstract] OR "alteplase"[Title/Abstract] OR "rtPA"[Title/Abstract] OR "tPA"[Title/Abstract] OR "tenecteplase"[Title/Abstract]	89769
	#5	#1 AND #2 AND #3 AND #4	504
Embase	#1	'fibrinolytic therapy'/exp	31259
	#2	fibrinolytic therapy:ab,ti OR ' fibrinolytic treatment ':ab,ti OR ' thrombolytic therapy ':ab,ti OR ' thrombolytic treatment ':ab,ti OR 'thrombolysis'/exp OR 'intravenous thrombolysis':ab,ti OR ivt:ab,ti OR 'bridging therapy':ab,ti OR 'bridging thrombolysis':ab,ti	93358
	#3	#1 OR #2	107470
	#4	' endovascular surgery'/exp	56040
	#5	'endovascular surgery':ab,ti OR 'endovascular procedure':ab,ti OR 'endovascular procedures':ab,ti OR 'surgery, endovascular':ab,ti	8366
	#6	#4 OR #5	58735
	#7	'carotid artery'/exp	152482
	#8	'carotid artery':ab,ti OR 'carotid arteries':ab,ti OR 'carotid artery diseases':ab,ti OR 'carotid artery stenosis':ab,ti OR 'carotid artery occlusion':ab,ti	112056

	#9	#7 OR #8	200657
	#10	'tandem lesion':ab,ti OR 'tandem lesions':ab,ti OR 'tandem occlusion':ab,ti OR 'tandem occlusions':ab,ti OR 'tandem stenosis':ab,ti OR 'tandem stenoses':ab,ti OR 'tandem pathology':ab,ti OR 'cervical carotid occlusion':ab,ti OR 'cervical carotid stenosis':ab,ti OR 'extracranial carotid occlusion':ab,ti OR 'extracranial carotid stenosis':ab,ti OR 'extracranial internal carotid artery':ab,ti OR 'cervical internal carotid artery':ab,ti OR 'internal carotid artery occlusion':ab,ti OR 'internal carotid artery stenosis':ab,ti OR 'carotid artery disease'/exp OR 'carotid artery stenosis'/exp OR 'carotid artery obstruction'/exp OR 'carotid artery thrombosis'/exp	98767
	#11	#3 AND #6 AND #9 AND #10	141
Cochrane	#1	MeSH descriptor: [Stroke] explode all trees	97404
	#2	MeSH descriptor: [Brain Ischemia] explode all trees	8816
	#3	("ischemic stroke" OR "ischaemic stroke" OR "acute ischemic stroke" OR "acute ischaemic stroke" OR "cerebral infarction" OR "large vessel occlusion" OR LVO):ti,ab,kw	21146
	#4	#1 OR #2 OR #3	100695
	#5	("tandem lesion" OR "tandem lesions" OR "tandem occlusion" OR "tandem occlusions" OR "tandem stenosis" OR "tandem stenoses" OR "tandem pathology" OR "tandem cervical"):ti,ab,kw	103
	#6	("cervical carotid occlusion" OR "cervical carotid stenosis" OR "extracranial carotid occlusion" OR "extracranial carotid stenosis" OR "cervical internal carotid artery" OR "extracranial internal carotid artery" OR "internal carotid artery occlusion" OR "internal carotid artery stenosis"):ti,ab,kw	374
	#7	MeSH descriptor: [Carotid Arteries] explode all trees	2171
	#8	MeSH descriptor: [Carotid Stenosis] explode all trees	2557
	#9	#5 OR #6 OR #7 OR #8	4462
	#10	MeSH descriptor: [Thrombectomy] explode all trees	3113
	#11	MeSH descriptor: [Endovascular Procedures] explode all trees	2545
	#12	("thrombectomy" OR "mechanical thrombectomy" OR "endovascular treatment" OR "endovascular therapy" OR "endovascular reperfusion" OR "endovascular intervention" OR EVT OR MT OR "stent retriever" OR "aspiration thrombectomy" OR "carotid artery stenting" OR "carotid stenting" OR stenting):ti,ab,kw	15669
	#13	#10 OR #11 OR #12	17016
	#14	MeSH descriptor: [Thrombolytic Therapy] explode all trees	4252
	#15	MeSH descriptor: [Tissue Plasminogen Activator] explode all trees	4204
	#16	("thrombolysis" OR "intravenous thrombolysis" OR IVT OR "bridging therapy" OR "bridging thrombolysis" OR alteplase OR rtPA OR tPA OR tenecteplase):ti,ab,kw	10441
	#17	#14 OR #15 OR #16	13091
	#18	#4 AND #9 AND #13 AND #17	116

Fig S1 PRISMA flow diagram of study search and selection for the meta-analysis

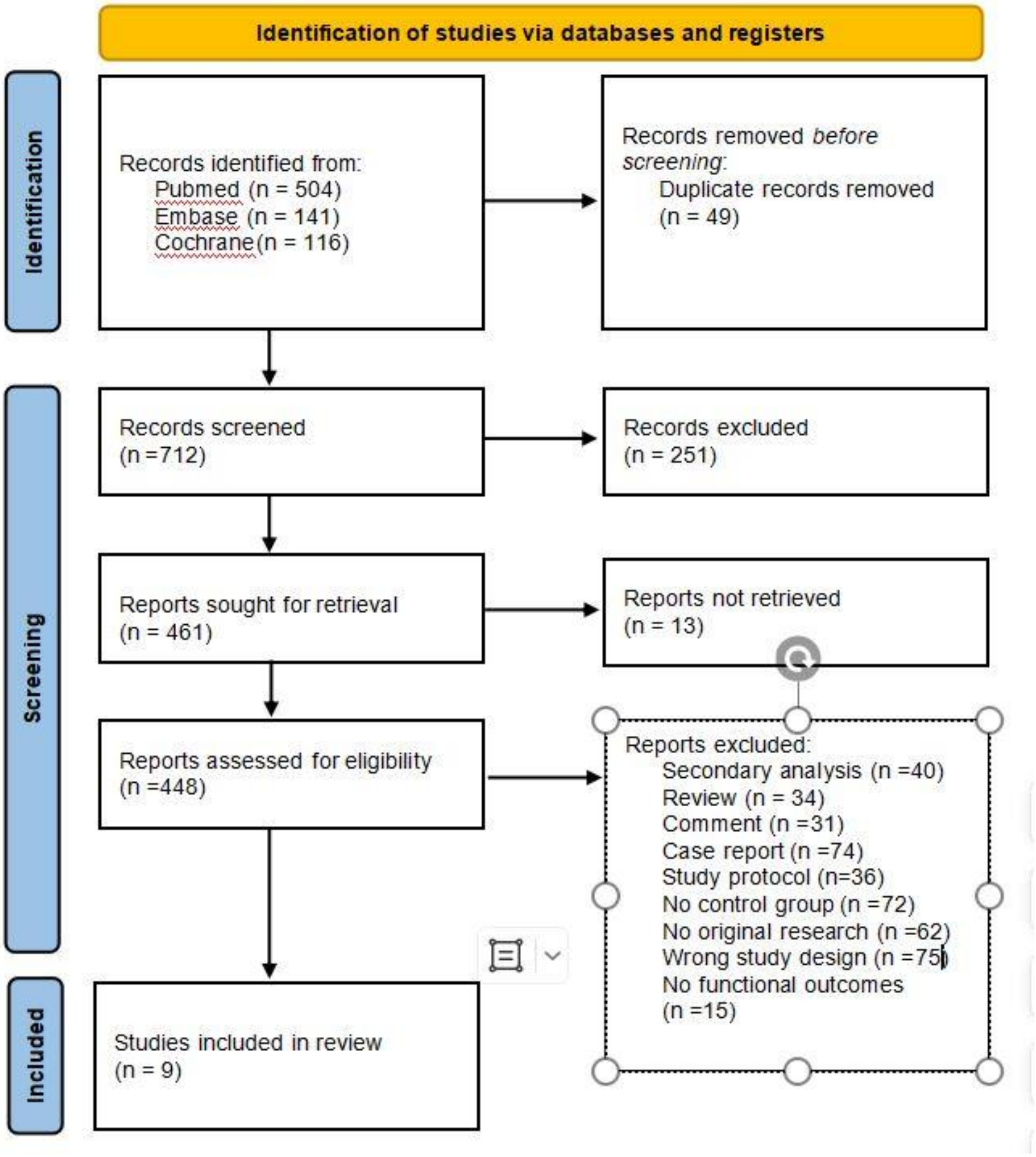

Table S2: Definitions of tandem lesions across included studies

Study	Extracranial lesion	Intracranial lesion	Stenosis threshold
Anadani 2022	Extracranial carotid artery steno-occlusive disease	Ipsilateral intracranial internal carotid artery (ICA) or M1/M2 segments of the middle cerebral artery(MCA)	Extracranial ICA stenosis ＞70%, or occlusion
Grigoryan,2016	Extracranial carotid artery steno-occlusive disease	Ipsilateral intracranial ICA、MCA(M1/M2) or anterior cerebral artery(ACA)	Not clearly specified
Heck,2015	Extracranial carotid artery steno-occlusive disease	Ipsilateral intracranial ICA、MCA(M1/M2)	Not clearly specified
Marnat,2021	Extracranial carotid artery steno-occlusive disease	Ipsilateral intracranial ICA、MCA(M1/M2)	Not clearly specified
Mujanovic,2024	Extracranial carotid artery steno-occlusive disease	Ipsilateral intracranial target lesion	Extracranial ICA stenosis ≥90%, or occlusion
Pikija,2019	Extracranial carotid artery steno-occlusive disease	Ipsilateral intracranial ICA、MCA	Not clearly specified
Rodriguez-Calienes,2023	Extracranial carotid artery steno-occlusive disease	Ipsilateral intracranial ICA、MCA(M1/M2) or ACA	Not clearly specified
Sallustio,2017	Extracranial carotid artery steno-occlusive disease	Ipsilateral intracranial ICA、MCA(M1/M2)	Not clearly specified
Sanak,2023	Extracranial carotid artery steno-occlusive disease	Anterior-circulation intracranial large-vessel occlusion	Not clearly specified

Fig S2 Forest plots comparing IVT combined with EVT with EVT alone across Functional and safety outcomes.

Fig S2A Forest plots of functional independence (mRS score, 0-2) at 90 days

Study or Subgroup	IVT combined with EVT Events	IVT combined with EVT Total	EVT alone Events	EVT alone Total	Weight	Risk Ratio M-H, Random, 95% CI
Anadani,2022	211	380	99	222	45.5%	1.25 [1.05, 1.48]
Grigoryan,2016	18	40	24	60	6.3%	1.13 [0.71, 1.79]
Heck,2015	4	7	8	16	2.1%	1.14 [0.51, 2.56]
Marnat,2021	57	91	22	46	11.6%	1.31 [0.93, 1.84]
Mujanovic,2024	21	33	14	30	6.3%	1.36 [0.86, 2.16]
Pikija,2019	18	37	2	16	0.8%	3.89 [1.02, 14.83]
Rodriguez-Calienes,2023	59	218	73	294	15.5%	1.09 [0.81, 1.46]
Sallustio,2017	14	39	9	33	2.8%	1.32 [0.66, 2.64]
Sanak,2022	72	131	21	63	9.2%	1.65 [1.13, 2.42]
**Total (95% CI)**		**976**		**780**	**100.0%**	**1.27 [1.13, 1.42]**
Total events	474		272			

Heterogeneity: Tau² = 0.00; Chi² = 6.06, df = 8 (P = 0.64); I² = 0%
Test for overall effect: Z = 4.02 (P < 0.0001)

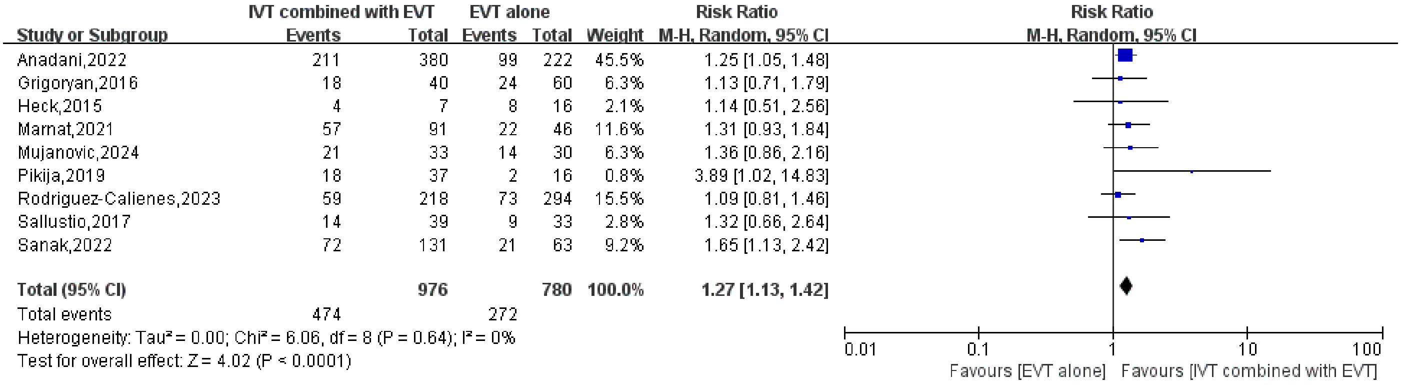

Fig S2B Forest plots of TICI ≥2b or3

Study or Subgroup	IVT combined with EVT Events	IVT combined with EVT Total	EVT alone Events	EVT alone Total	Weight	Risk Ratio M-H, Random, 95% CI
Anadani,2022	301	380	155	222	24.3%	1.13 [1.03, 1.25]
Heck,2015	6	7	11	16	1.9%	1.25 [0.80, 1.95]
Marnat,2021	78	94	32	50	6.8%	1.30 [1.03, 1.63]
Mujanovic,2024	33	33	26	30	13.7%	1.15 [0.99, 1.34]
Rodriguez-Calienes,2023	194	218	253	294	37.9%	1.03 [0.97, 1.10]
Sanak,2022	116	131	50	63	15.3%	1.12 [0.97, 1.28]
**Total (95% CI)**		**863**		**675**	**100.0%**	**1.11 [1.04, 1.18]**
Total events	728		527			

Heterogeneity: Tau² = 0.00; Chi² = 6.83, df = 5 (P = 0.23); I² = 27%
Test for overall effect: Z = 3.17 (P = 0.002)

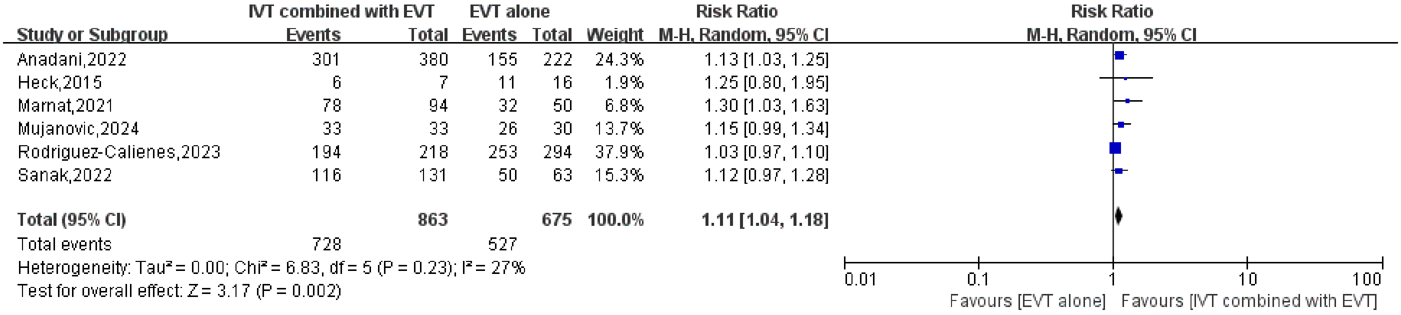

Fig S2C Forest plots of mortality at 90 days

Study or Subgroup	IVT combined with EVT Events	IVT combined with EVT Total	EVT alone Events	EVT alone Total	Weight	Risk Ratio M-H, Random, 95% CI
Anadani,2022	40	380	35	222	36.5%	0.67 [0.44, 1.02]
Marnat,2021	4	91	6	30	4.6%	0.22 [0.07, 0.73]
Mujanovic,2024	5	33	6	30	5.6%	0.76 [0.26, 2.23]
Rodriguez-Calienes,2023	29	218	50	294	36.5%	0.78 [0.51, 1.19]
Sanak,2022	19	131	14	63	16.9%	0.65 [0.35, 1.22]
**Total (95% CI)**		**853**		**639**	**100.0%**	**0.67 [0.52, 0.87]**
Total events	97		111			

Heterogeneity: Tau² = 0.00; Chi² = 3.91, df = 4 (P = 0.42); I² = 0%
Test for overall effect: Z = 3.02 (P = 0.003)

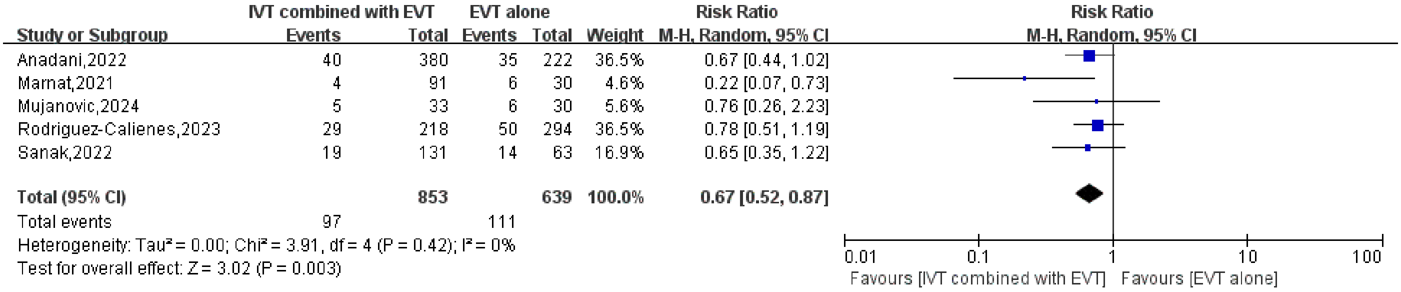

Fig S2D Forest plots of symptomatic intracranial hemorrhages (sICH)

Study or Subgroup	IVT combined with EVT Events	IVT combined with EVT Total	EVT alone Events	EVT alone Total	Weight	Risk Ratio M-H, Random, 95% CI
Anadani,2022	27	380	18	222	35.8%	0.88 [0.49, 1.55]
Heck,2015	0	7	5	16	2.6%	0.19 [0.01, 3.08]
Marnat,2021	4	94	7	47	12.5%	0.29 [0.09, 0.93]
Mujanovic,2024	11	33	14	30	32.9%	0.71 [0.39, 1.32]
Rodriguez-Calienes,2023	4	37	5	33	11.7%	0.71 [0.21, 2.44]
Sallustio,2017	4	94	7	47	0.0%	0.29 [0.09, 0.93]
Sanak,2022	8	131	1	63	4.6%	3.85 [0.49, 30.10]
**Total (95% CI)**		**682**		**411**	**100.0%**	**0.72 [0.46, 1.12]**
Total events	54		50			

Heterogeneity: Tau² = 0.06; Chi² = 6.24, df = 5 (P = 0.28); I² = 20%
Test for overall effect: Z = 1.45 (P = 0.15)

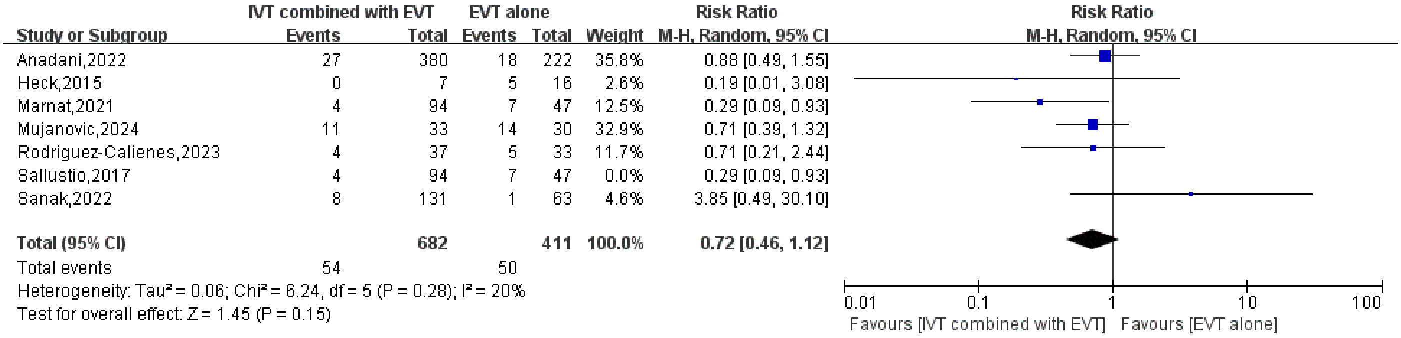

Note: Risk ratios (RRs) with 95% confidence intervals (CIs) were pooled using the Mantel-Haenszel random-effects model. Study weights were calculated according to the Mantel-Haenszel method under the random-effects assumption. Squares indicate study-specific effect estimates, with the size of each square proportional to the study weight; horizontal lines indicate 95% CIs; and diamonds represent pooled effect estimates. Event counts, total sample sizes, and study weights are shown in each forest plot.
